# Unravelling the Significance of Phosphoenolpyruvate Carboxylase in Phosphate Starvation Responses

**DOI:** 10.1111/pce.70204

**Published:** 2025-09-24

**Authors:** Jesús Pérez‐López, Clara de la Osa, Jacinto Gandullo, Nora Gigli‐Bisceglia, Inmaculada Coleto, Ana Belén Feria, Cristina Echevarría, Christa Testerink, Daniel Marino, Sofía García‐Mauriño, José A. Monreal

**Affiliations:** ^1^ Departamento de Biología Vegetal y Ecología Facultad de Biología, Universidad de Sevilla Seville Spain; ^2^ Laboratory of Plant Physiology, Plant Sciences Group Wageningen University and Research Wageningen the Netherlands; ^3^ Laboratory of Plant Stress Resilience, Institute of Environmental Biology Utrecht University Utrecht the Netherlands; ^4^ Departamento de Biología Vegetal y Ecología Facultad de Ciencia y Tecnología, Universidad del País Vasco/Euskal Herriko Unibertsitatea (UPV/EHU) Leioa Spain

**Keywords:** calcium phosphate, phosphate acquisition efficiency (PAE), phosphate starvation responses (PSR), phosphoenolpyruvate carboxylase (PEPC), root exudates, *Sorghum bicolor*

## Abstract

Low phosphate availability is a major concern for agriculture. Plants develop a plethora of responses to improve phosphate acquisition, known as phosphate starvation responses (PSR). Among them, the induction of phosphoenolpyruvate carboxylase (PEPC) has been described in many plants. However, most studies have been conducted in the absence of phosphate, thus the real impact of PEPC in PSR is missing as there is no phosphate to take up. In this study, we used modified sorghum plants silenced in the main PEPC isozyme in roots, SbPPC3, and analyzed the role of PEPC in the presence of insoluble calcium phosphate (PCa), showing a phosphate starvation phenotype in silenced but not in WT plants. Interestingly, root exudation of citrate was not reduced in silenced plants, probably due to a higher citrate synthase activity, but it was reduced for succinate, another compound with phosphate solubilisation capacity. Finally, silenced plants accumulated less P in roots with PCa, leading to a reduced phosphate acquisition efficiency (PAE). Our results show, for the first time, the actual role of PEPC in phosphate solubilisation through succinate exudation, proposing PPC3 as a specific target to improve PAE in plants.

## Summary statement

In *Sorghum Bicolor*, the non‐photosynthetic phosphoenolpyruvate carboxylase 3 (SbPPC3) protein participates in phosphate starvation responses (PSR) and is crucial for succinate exudation by roots, affecting phosphate solubilisation and phosphate acquisition efficiency (PAE).

## 1 Introduction

Phosphorus is an essential macronutrient for all plants and, together with N and K, the main nutrients added as fertilisers due to the low amounts usually present in agricultural soils. Phosphate deficiency inhibits crop productivity and affects about 70% of arable land (Estelle and Somerville 1987). Plants preferentially absorb phosphorus from the soil in its fully oxidised anionic form inorganic phosphate H_2_PO_4_^−^ and HPO_4_^2−^ (Pi) (O'Leary et al. 2011). Despite its importance, Pi is one of the least available macronutrients in many terrestrial and aquatic environments. Its high tendency to form insoluble complexes with metal cations such as Al^3+^, Ca^2+^ and Fe^3+^, its existence in organic forms (Po), and its low rates of diffusion make Pi the least readily available nutrient in the rhizosphere (Raghothama 2000). Reserves of rock Pi, the major source of phosphate fertilisers, are scarce and located in few countries, with the world reserves expected to be exhausted by the end of this century (Blanco 2011; Dissanayaka et al. 2021). About 80% of Pi applied as fertiliser is not available for plant use because it binds to metal cations becoming insoluble, is converted into Po by soil microbes, or runs off causing important environmental problems. Therefore, phosphate starvation is a major problem for agriculture and urges to find solutions to improve the use and absorption of this element by plants. In the lack of phosphate, plants develop a myriad of responses to improve phosphate acquisition efficiency (PAE), collectively known as phosphate starvation responses (PSR). Most common PSR are upregulation of high‐affinity phosphate transporters at the root plasma membrane (*PHT1* gene family), acidification of the rhizosphere (Moorby et al. 1988), exudation of Pi‐mobilising compounds such as organic acid anions (OAs), nucleases and acid phosphatases (APases), alterations of the root system architecture (RSA) to forage phosphate from the topsoil (reduction of primary root growth and enhanced lateral root formation, together with an increase in the number and size of root hairs), and symbiotic association with mycorrhiza (Dissanayaka et al. 2021).

Root exudation of OAs, mainly malate and citrate (together termed “carboxylates”), mobilises Pi sorbed to Al^3+^ or Fe^2+^ in acidic soils, and to Ca^2+^ in alkaline soils, increasing soil solution Pi concentrations by up to 1000‐fold (Plaxton and Tran 2011). OAs show high affinity for metal cations, displacing phosphate from insoluble complexes through ligand exchange, making P available for plant uptake (de Bang et al. 2021). In addition, exudation of OAs can recruit beneficial soil bacteria (Rudrappa et al. 2008), that may solubilise phosphate. Under phosphate starvation, some species translocate up to 25% of photosynthetically fixed CO_2_ to the roots to support the synthesis and exudation of carboxylates into the rhizosphere (Canarini et al. 2019). This exudation of carboxylates is accompanied by the upregulation of phosphoenolpyruvate carboxylase (PEPC) and its phosphorylation state (Feria et al. 2016; Pérez‐López et al. 2023a), malate dehydrogenase (MDH) and citrate synthase (CS) (Plaxton and Tran 2011). The proposed roles for PEPC in this context are, first, to provide flexibility to PEP metabolism when ADP levels are limiting through, together with MDH and NAD‐malic enzyme (NAD‐ME), creating a glycolytic bypass of the cytosolic pyruvate kinase (PKc) while recycling the PEPC by‐product Pi for its reassimilation (Plaxton and Podestá 2006); and second, to rise the synthesis of citrate and malate in roots that can be exudated afterwards to the rhizosphere to solubilise insoluble forms of phosphate (O'Leary et al. 2011).

PEPC (EC 4.1.1.31) is a cytosolic enzyme catalysing the irreversible β‐carboxylation of phosphoenolpyruvate (PEP), using bicarbonate as substrate, to yield oxaloacetate (OAA) and Pi (Chollet et al. 1996). PEPC has been extensively studied in C_4_ and CAM plants, where it carries out the initial fixation of atmospheric CO_2_. In leaves from C_4_ plants, OAA can either be reduced to malate by MDH in the mesophyll chloroplast, or converted to aspartate by aspartate aminotransferase (AAT) in the cytosol, and transported to the bundle sheath cells (Kanai and Edwards 1999). In addition, PEPC participates in other non‐photosynthetic metabolic contexts where malate or aspartate may have a role, such as C/N interactions and anaplerotic flux, stomatal opening control, root excretion, seed development and germination, or in responses to stress (Doubnerová and Ryšlavá 2011; O'Leary et al. 2011).

Sorghum (*Sorghum bicolor*) is a C_4_ cereal and the 5th most important crop worldwide, being a staple crop for over 500 million people, mainly in Africa and Asia, and used for human food, livestock feed, renewable industrial materials, and biofuels (Zhao and Dahlberg 2019). Sorghum is considered to be moderately tolerant to abiotic stresses such as drought or salinity (Mansour et al. 2021; de la Osa et al. 2022). PEPC protein is composed of a multigene family, with 6 members in sorghum (Sb*PPC1‐6*) (Paterson et al. 2009) with different patterns of expression and location. SbPPC1 is the isozyme participating in C_4_ photosynthesis, and the major PEPC protein in leaves (Shenton et al. 2006). SbPPC2‐5 are C_3_‐type PEPCs carrying out the non‐photosynthetic functions described above, although the specific contribution of each isozyme remains mostly unknown. SbPPC2 displays similar expression level in all tissues (Shenton et al. 2006). SbPPC3 is the most abundant PEPC isozyme in seeds (Ruiz‐Ballesta et al. 2016) and roots, where is induced in response to ammonium stress (Arias‐Baldrich et al. 2017; Marín‐Peña et al. 2024), salinity (de la Osa et al. 2022), or aluminum and cadmium toxicity (Pérez‐López et al. 2023b). SbPPC4 seems to participate during early seed development (Ruiz‐Ballesta et al. 2016), and SbPPC5 is of unknown function and almost absent in all tissues analyzed to date. SbPPC1‐5 are referred as plant‐type PEPCs (PTPCs), in opposition with SbPPC6, referred as bacterial‐type PEPC (BTPC) (O'Leary et al. 2011).

PEPC activity is regulated in vivo by a set of different posttranslational modifications (PTMs). Phosphorylation of PTPCs changes the kinetic properties of the enzyme by rising its maximal reaction rate, affinity to PEP, and its activation by glucose 6‐P, and decreasing the inhibition of the enzyme by l‐malate (Echevarria et al. 1994; Takahashi‐Terada et al. 2005). PEPC is phosphorylated by a dedicated Ca^2+^‐independent serine/threonine kinase known as PEPC kinase (PPCK) (Echevarria and Vidal 2003). The phosphorylation state of PEPC can be easily estimated by the IC_50_ (l‐malate concentration causing a 50% reduction of PEPC activity at pH 7.3), with higher IC_50_ values indicating higher phosphorylation of the protein in vivo (Echevarría et al. 1990). In addition, sorghum PEPCs may be regulated by other PTMs such as mono‐ubiquitination (Ruiz‐Ballesta et al. 2014), S‐nitrosylation (Monreal et al. 2013), carbonylation (Baena et al. 2017), or binding to anionic phospholipids (Monreal et al. 2010; Gandullo et al. 2019).

Although PEPC has been widely described to be induced in response to phosphate starvation in many plants (O'Leary et al. 2011; Dissanayaka et al. 2021), most of the research on the role of PEPC in this context has been carried out in the absence or in very low amounts of this nutrient in its readily available soluble form (NaH_2_PO_4_ or KH_2_PO_4_). Thus, the actual importance of PEPC in a context of low Pi availability in the soil, and how successful is this response, is missing, as the plant is not able to uptake higher amounts of Pi no matter what the responses are. To test the significance of PEPC in a situation where P is present but insoluble, a common situation in soils, we have grown sorghum plants hydroponically not only in the absence of phosphate (‐P treatment), but also in the presence of insoluble phosphate in the form of calcium phosphate (Ca_3_(PO_4_)_2_; PCa treatment). Moreover, by silencing the predominant *PPC* gene in roots, Sb*PPC3*, we demonstrate the actual importance of this PEPC isozyme to cope with insoluble phosphate, a common form of phosphate found in soils.

## 2 Results

### 2.1 Phosphate Starvation Rises PEPC Activity and Quantity in Sorghum Roots

**Figure 1:**
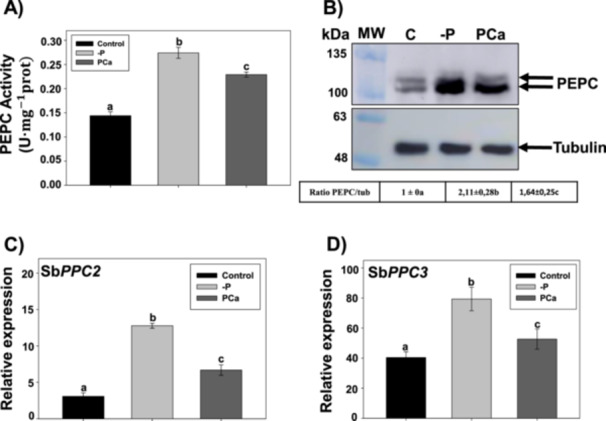
Phosphate starvation induces *PPC* gene expression and PEPC activity and protein in roots from WT plants. Plants were grown hydroponically with nitrate‐type nutrient solution (Hewitt) with 1.2 mM NaH_2_PO_4_ (soluble phosphate, Control), without phosphate (‐P), or with 0.6 mM Ca_3_(PO_4_)_2_ (insoluble phosphate, PCa), for 3 weeks, as described in Section 5. (A) PEPC activity in sorghum roots. (B) Immunodetection of PEPC and tubulin proteins in sorghum root extracts. For analysis of protein levels, 50 µg per lane of total proteins were loaded for SDS‐PAGE and transferred onto nitrocellulose membranes for blotting using anti‐PTPC and anti‐tubulin antibodies, as described in Section 5. Arrows indicate the ubiquitinated PEPC (upper), the non‐ubiquitinated PEPC (lower), and tubulin. Signal intensities were measured using ImageStudioTM Lite software (LI‐COR Biosciences). Table indicates ratio PEPC/tubulin (mean SE, *n* = 5). (C) Relative transcript abundance of Sb*PPC2* and (D) Sb*PPC3* in sorghum roots. For each gene, data are normalised to the transcript abundance in WT leaves in control conditions. Bars in (A), (C) and (D) indicate mean ± SE (*n* = 4), where each biological sample is composed of a pool of three individual roots growing in the same pot. Columns that do not have a common letter are significantly different by Duncan's multiple range test (*p* < 0.05).

PEPC is induced in response to phosphate starvation in many plants as a part of the PSR. To test whether this was also the case for sorghum, we grew plants hydroponically with three different regimes of phosphate: soluble phosphate in the form of NaH_2_PO_4_ (control), total absence of phosphate (‐P), and insoluble phosphate as Ca_3_(PO_4_)_2_ (PCa). As expected, PEPC activity (Figure 1A) and immuno‐reactive PTPC polypeptides (Figure 1B) were increased about twofold in the absence of phosphate (‐P) in roots from WT plants. In the presence of insoluble phosphate (PCa), PEPC activity and quantity were induced compared to control conditions, but to a lesser extent compared to ‐P. A similar response to phosphate treatments was observed when analyzing the pattern of expression of Sb*PPC2* (Figure 1C) and Sb*PPC3* genes (Figure 1D), the main *PPC* genes in roots. Transcripts of the other non‐photosynthetic *PPC* genes (Sb*PPC4‐6*) were not detected in any condition in this tissue, as for the case of the photosynthetic gene Sb*PPC1*. In leaves, the expression of Sb*PPC2* and Sb*PPC3* were importantly induced in ‐P plants, whereas PCa induced a moderate, albeit not significant, increment in their expression. Notably, Sb*PPC3* expression was more induced in response to stress in leaves compared to Sb*PPC2*. However, this increase in gene expression by phosphate starvation was not translated into a rise in PEPC activity nor immune‐reactive polypeptide, presumably due to the predominant presence of the photosynthetic C_4_‐PEPC in leaves (Supporting Information S1: Figure S1). Sb*PPC1* expression was not affected by phosphate treatments and, as in the case of roots, Sb*PPC4‐6* expression was not detected.

### 2.2 PPC3 Is the Main PEPC Isozyme Implicated in Responses to Phosphate Starvation

**Figure 2:**
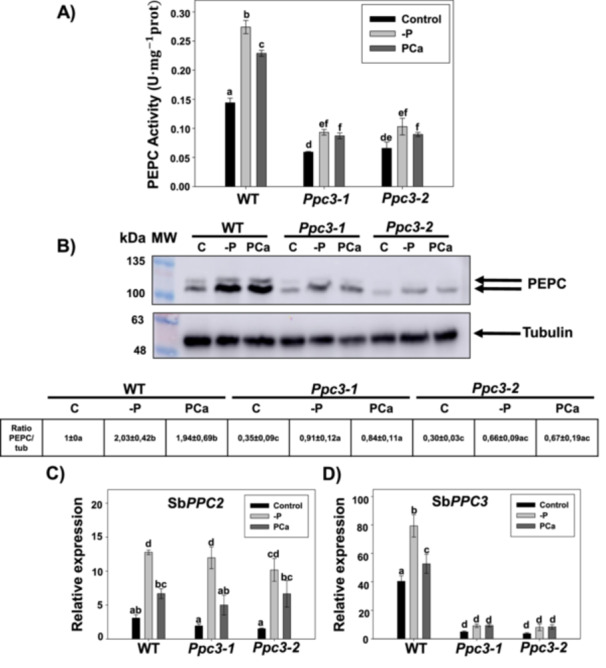
Effect of phosphate starvation in sorghum roots from WT and *Ppc3* plants. Plants were grown hydroponically as described in Figure 1 and in Section 5. (A) PEPC activity in sorghum roots. (B) Immunodetection of PEPC and tubulin proteins in sorghum root extracts. MW: molecular markers. Arrows indicate the ubiquitinated PEPC (upper) and the non‐ubiquitinated PEPC (lower), and tubulin. Table indicates ratio PEPC/tubulin (mean ± SE, *n* = 5). (C) Relative transcript abundance of Sb*PPC2* and (D) Sb*PPC3* in sorghum roots. For each gene, data are normalised to the transcript abundance in WT leaves in control conditions. Bars in (A), (C) and (D) indicate mean ± SE (*n* = 4), where each biological sample is composed of a pool of three individual roots growing in the same pot. Columns that do not have a common letter are significantly different by Duncan's multiple range test (*p* < 0.05). [Color figure can be viewed at wileyonlinelibrary.com]

To assess the relevance of non‐photosynthetic PTPCs in sorghum response to phosphate starvation, we took advantage of modified sorghum plants were Sb*PPC3* expression was silenced by RNAi (*Ppc3* lines) (de la Osa et al. 2022). We selected 2 independent lines with ~90% reduction in Sb*PPC3* expression in leaves and roots, lines *Ppc3‐1* and *Ppc3‐2* (de la Osa et al. 2022). The rise of PEPC activity and immuno‐reactive PTPC polypeptides induced by phosphate treatments was importantly reduced in roots from *Ppc3* plants (Figure 2A,B). When analyzing the expression of *PPC* genes, Sb*PPC2* was similarly induced in WT and *Ppc3* plants by phosphate treatments (Figure 2C), but not Sb*PPC3*, whose expression was barely affected by the treatments and remained silenced (Figure 2D). Hence, the small induction of PEPC activity detected in *Ppc3* lines in response to phosphate treatments (Figure 2A) might be due to the induction of Sb*PPC2*. As in WT plants, Sb*PPC4‐6* expression was not detected in any condition. These results show that the increase of PEPC activity upon phosphate starvation in roots is mainly due to increased *PPC3* expression and PPC3 activity. The lack of Sb*PPC3* transcripts is not compensated by other *PPC* genes as their expression in silenced lines is similar to WT plants. In leaves, the expression of Sb*PPC2* was induced by phosphate treatments in a similar manner in all plants, with higher increment by ‐P compared to PCa treatments (Supporting Information S1: Figure S2). As in roots, the effect of phosphate treatments on Sb*PPC3* expression found in WT leaves was abolished in silenced plants, but in this case it did not affect total PEPC activity nor immuno‐reactive PTPC due to the presence of photosynthetic PEPC, whose expression was not affected by any treatment regardless the genotype.

### 2.3 Silenced Plants Show Phosphate Starvation Symptoms in the Presence of Calcium Phosphate

**Figure 3:**
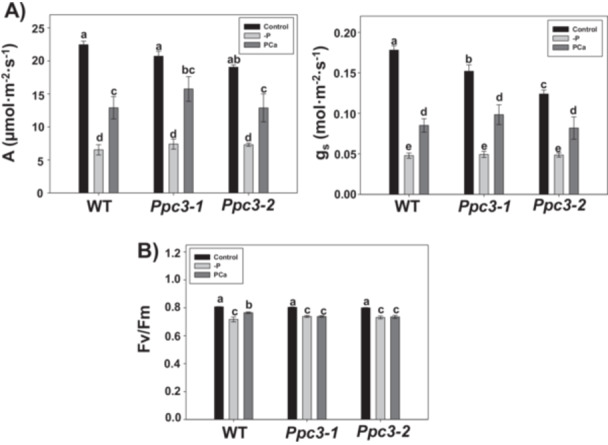
Effect of phosphate starvation on photosynthetic parameters in WT and *Ppc3* plants. After 3 weeks growing with phosphate treatments, photosynthetic parameters were analyzed in the youngest fully developed leaf of each plant, as indicated in Section 5. (A) Net photosynthetic rate (A, left panel) and stomatal conductance (g_s_, right panel). (B) Maximum quantum yield of PSII (Fv/Fm). Bars indicate mean ± SE (*n* = 7–9, one measure per plant). Columns that do not have a common letter are significantly different by Duncan's multiple range test (*p* < 0.05).

To analyze the effect of Sb*PPC3* silencing in plant behaviour under phosphate starvation, we analyzed several parameters affected by this stress in WT and *Ppc3* plants. As expected, phosphate treatments affected photosynthesis, reducing net photosynthetic rate (A) and stomatal conductance (g_s_), in a similar manner in WT and *Ppc3* plants (Figure 3A). As described in de la Osa et al. (2022), silenced lines showed lower g_s_ in control conditions compared to WT plants. In all plants, the drop in A and g_s_ was more pronounced with the total absence of phosphate (‐P) compared to the application of insoluble phosphate (PCa), suggesting a higher stress severity for ‐P treatment. Photosynthetic pigments chlorophylls (a + b) and carotenoids were not significantly affected by silencing or phosphate treatments (Supporting Information S1: Figure S3). However, the effect on the maximum quantum yield of PSII (Fv/Fm), an index that is reduced under stress conditions, was similar between WT and silenced plants only in ‐P treatments, but not in PCa, where WT showed higher values of Fv/Fm compared to ‐P, and *Ppc3* plants did not (Figure 3B). This indicates that silenced *Ppc3* lines are more stressed than WT plants when phosphate is insoluble.

**Figure 4:**
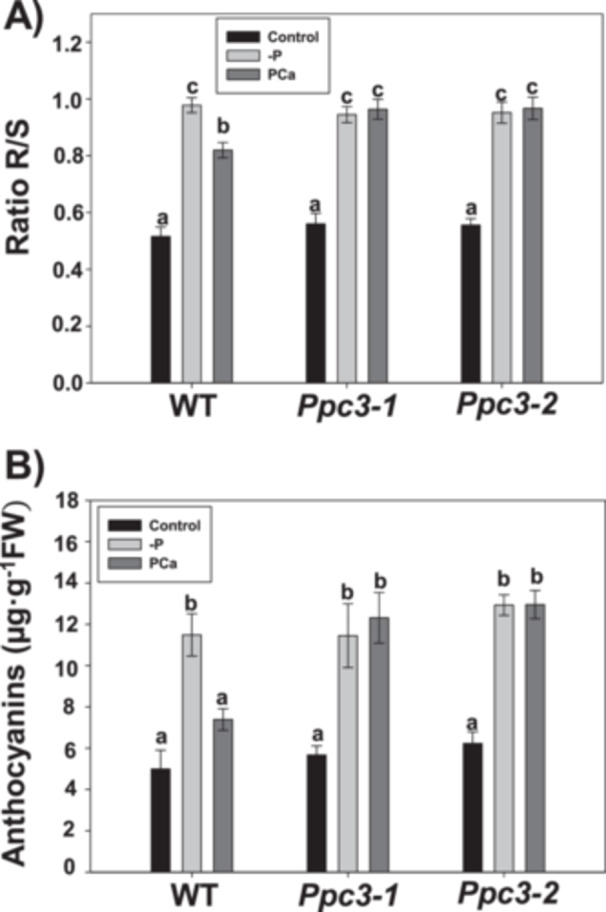
Phosphate starvation responses induced in WT and *Ppc3* plants. Three weeks after growing hydroponically under phosphate treatments, plants were harvested and phosphate starvation responses analyzed as described in Section 5. (A) Ratio root/shoot (R/S). (B) Anthocyanins in leaves. Bars indicate mean ± SE (*n* = 4–8). Columns that do not have a common letter are significantly different by Duncan's multiple range test (*p* < 0.05).

Under phosphate scarcity, plant invest more resources to develop roots instead of shoots (Jiang et al. 2007). In agreement, roots‐to‐shoots biomass ratio (R/S) increased in ‐P and PCa but in a different manner depending on the treatment and the sorghum line. Whereas in WT plants R/S was higher in ‐P treatment compared to PCa, in silenced plants R/S was similar in both ‐P and PCa treatments (Figure 4A). As expected, phosphate starvation produced an important reduction in plant height and biomass, although in a similar extent in all plants (Supporting Information S1: Figure S4). Another common response to phosphate starvation is the accumulation of anthocyanins in leaves (de Bang et al. 2021). In ‐P, all genotypes presented higher levels of anthocyanins. In contrast, in PCa only *Ppc3* lines showed the increase in anthocyanin content, indicating that the stress degree experienced by these lines in PCa was higher compared to WT plants (Figure 4B). Altogether, these results indicate that, first, PCa treatment induces PSR, and second, these responses are more induced in *Ppc3* compared to WT plants, suggesting higher phosphate starvation in silenced plants.

**Figure 5:**
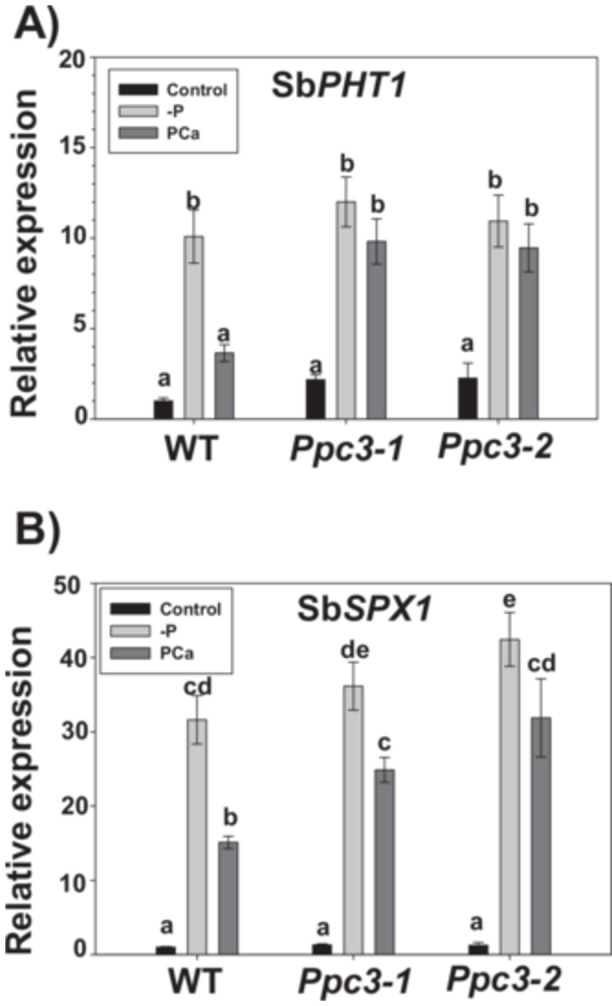
Molecular markers of phosphate starvation in roots from WT and *Ppc3* plants. Three weeks after growing hydroponically under phosphate treatments, roots were harvested and phosphate starvation molecular markers were analyzed by qPCR, as described in Section 5. (A) Sb*PHT1*. (B) Sb*SPX1*. For each gene, data are normalised to the transcript abundance in WT roots in control conditions. Bars indicate mean ± SE (*n* = 4), where each biological replicate correspond to roots from three plants in the same pot. Columns that do not have a common letter are significantly different by Duncan's multiple range test (*p* < 0.05).

Next, we analyzed molecular markers for PSR, namely SbPHT1 and SbSPX1. SbPHT1 (Sb01g046890) is a high affinity phosphate transporter (Walder et al. 2015), and SPX1 is a nuclear protein involved in phosphate starvation signalling (Wang et al. 2009). Both Sb*PHT1* and Sb*PX1* expression was highly induced in the absence of P (‐P), to a similar extent in all plants. However, the induction of these genes in WT plants in PCa was lower compared with ‐P. Interestingly, in silenced *Ppc3* plants the expression of both genes in PCa was higher compared to WT plants (Figure 5A). Altogether, the higher expression of molecular markers for phosphate starvation in roots, together with the photosynthetic, anatomical, and biochemical indicators described above, evidence that silenced plants are more stressed than WT plants in the presence of insoluble phosphate. Thus, underlining the importance of root PEPC for phosphate solubilisation.

### 2.4 Central Metabolism Is Affected by Phosphate Starvation

**Figure 6:**
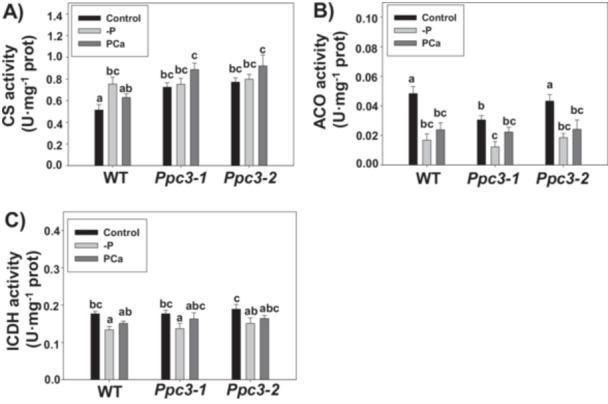
Effect of phosphate starvation on TCA enzymes in roots from WT and *Ppc3* plants. Three weeks after growing hydroponically under phosphate treatments, plants were harvested and TCA enzymatic activities from roots analyzed as described in Section 5. (A) Citrate synthase (CS), (B) Aconitase (ACO) and (C) Isocitrate deshydrogenase (ICDH). Bars indicate mean ± SE (*n* = 3–7). Columns that do not have a common letter are significantly different by Duncan's multiple range test (*p* < 0.05).

Due to the pivotal role of PEPC in plant central metabolism, the effect of phosphate starvation on enzymatic activities and metabolites related to the TCA was analyzed in roots from WT and *Ppc3* plants. The most important changes in enzymatic activities were found in enzymes implicated in citrate metabolism, namely CS, aconitase (ACO) and isocitrate dehydrogenase (ICDH). CS activity was induced in WT roots in the absence of phosphate (‐P). Silenced plants showed higher CS activity in roots in control conditions compared to WT plants (de la Osa et al. 2022), but it was not increased in ‐P, showing similar levels to WT ‐P and *Ppc3* control. Importantly, in the presence of PCa, CS activity was higher in silenced compared to WT roots (Figure 6A). ACO and ICDH were reduced in all plants only in ‐P treatment (Figure 6B,C). NAD‐ME, PK and MDH were almost unaffected by the treatment or the genotype in plant roots (Supporting Information S1: Figure S5). In leaves, the absence of P (‐P) reduced CS activity only in silenced lines, whereas both phosphate treatments (‐P and PCa) reduced ICDH activity in all lines. NADP‐ME, NAD‐ME, PK and MDH were mostly unaffected. No differences were detected between WT and silenced plants (Supporting Information S1: Figure S6).

**Figure 7:**
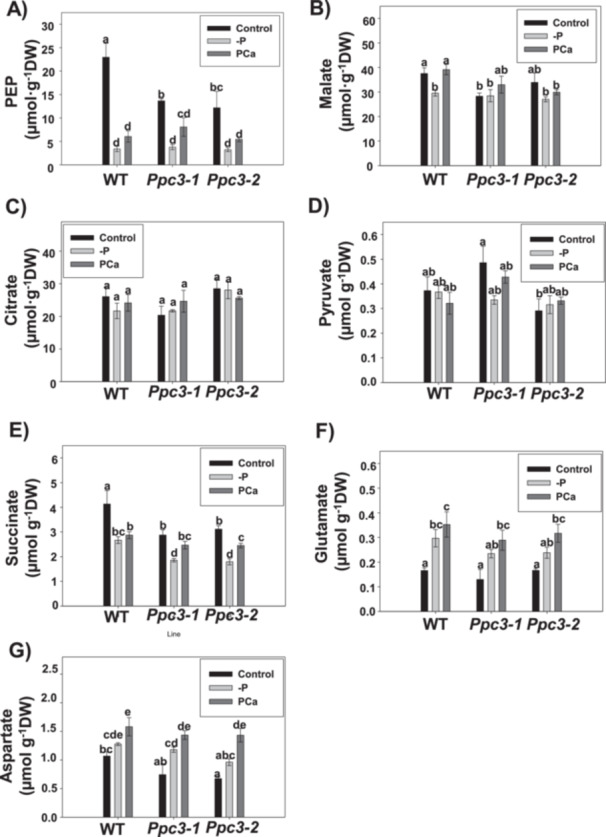
Effect of phosphate starvation on organic acid accumulation in WT and *Ppc3* roots. Organic acids were analyzed in lyophilised roots by ion chromatography, as described in Section 5. (A) Phosphoenolpyruvate (PEP), (B) Malate, (C) Citrate, (D) Pyruvate, (E) Succinate, (F) Glutamate and (G) Aspartate. Bars indicate mean ± SE (*n* = 4–5). Columns that do not have a common letter are significantly different by Duncan's multiple range test (*p* < 0.05).

The most abundant OAs related to the TCA found in roots were PEP, malate and citrate, whereas pyruvate and succinate were found in lower amounts, as well as amino acids glutamate and aspartate (Figure 7). PEP was drastically reduced in roots from all plants in response to phosphate treatments. Notably, silenced roots showed lower amounts of this compound than WT roots in control conditions (Figure 7A). Malate levels were reduced in WT roots without phosphate (‐P), while its level was similar in control and PCa conditions. In silenced roots, malate accumulation was barely affected by phosphate treatments (Figure 7B). Citrate levels were similar among all plants and treatments (Figure 7C). Altogether, these results indicate that the changes in PEPC activity or in enzymes related to citrate metabolism (CS, ACO and ICDH), together with the low levels of PEP observed in phosphate treatments, do not account for an accumulation of citrate or malate in roots in response to phosphate starvation in sorghum. The level of pyruvate in roots was ~70‐fold lower than the level of citrate or malate, and was not affected in any line or treatment (Figure 7D). However, the level of succinate, although lower than the level of citrate or malate in roots (~7‐fold in WT control), was reduced in response to phosphate treatments, and silenced lines accumulated less succinate than WT plants in control and ‐P treatments (Figure 7E). Finally, amino acids glutamate (Figure 7F) and aspartate (Figure 7G) also showed lower levels in roots compared to malate or citrate, but were accumulated in response to phosphate treatments in all lines.

### 2.5 Silencing of SbPPC3 Alters OAs Exudation and PAE

**Figure 8:**
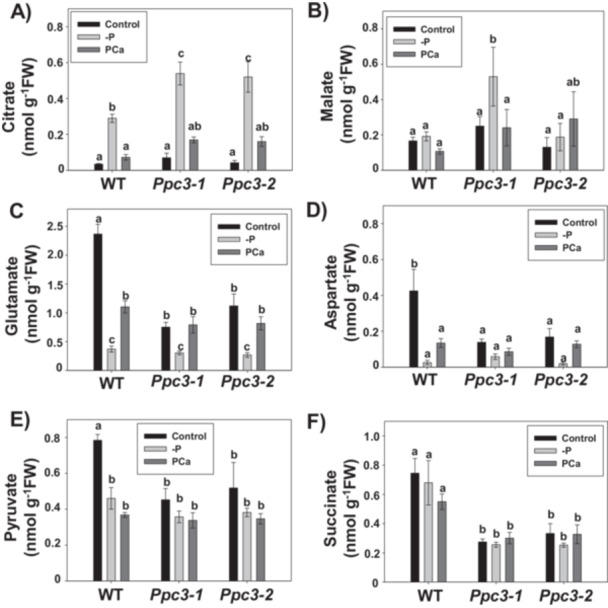
Organic acid exudation in WT and *Ppc3* plants under phosphate treatments. Exudates from sorghum roots were collected, lyophilised and analyzed as described in Section 5. (A) Citrate, (B) Malate, (C) Glutamate, (D) Aspartate, (E) Pyruvate and (F) Succinate. Bars indicate mean ± SE (*n* = 3–5). Columns that do not have a common letter are significantly different by Duncan's multiple range test (*p* < 0.05).

OAs can be exudated to the rhizosphere to increase phosphate mobilisation in the soil (Dissanayaka et al. 2021). Thus, the changes detected previously in enzymatic activities may induce changes in secretion of OAs by roots instead than in their accumulation in roots. Therefore, we analyzed the amount of OAs exudated by WT and *Ppc3* roots under phosphate treatments (Figure 8). Citrate was the sole OA for which exudation was clearly induced in response to total phosphate starvation (‐P), but not to PCa, in all plants (Figure 8A). Surprisingly, citrate exudation in silenced *Ppc3* plants was even higher than in WT plants in ‐P conditions. Malate exudation increased only in *Ppc3‐1*, but not in WT or *Ppc3‐2* plants, in response to phosphate starvation (‐P) (Figure 8B). Glutamate exudation was reduced in ‐P, and partially recovered in PCa in all plants (Figure 8C). Aspartate and pyruvate were exudated in lower amounts in ‐P and PCa treatments in WT plants but not in silenced plants, where the level of these two OAs were lower compared to WT plants and scarcely affected by phosphate treatments (Figure 8D,E). Finally, succinate exudation was barely affected by phosphate treatments in any plant (Figure 8G), albeit its exudation in silenced plants was smaller than in WT plants in any condition analyzed, constituting the major difference in OAs exudation between WT and silenced plants in PCa treatment. Interestingly, silenced plants exudated lower levels of glutamate, aspartate, pyruvate, and succinate in control conditions compared to WT plants. When analyzing the relative contribution of each OA to the total amount of exudates, glutamate was the major OA exudated in control conditions in all lines. In the absence of P (‐P), the most abundant OA was succinate in WT plants, but citrate in silenced lines. In PCa we found an intermediate situation: glutamate and succinate for WT plants, and glutamate, pyruvate, succinate and malate for *Ppc3* lines. Therefore, although citrate is more exudated by silenced plants compared to WT plants in response to phosphate starvation, the importance of other OAs such as glutamate and succinate, which secretion is reduced in *Ppc3* plants, has to be taken into account in the phosphate solubilisation process.

**Figure 9:**
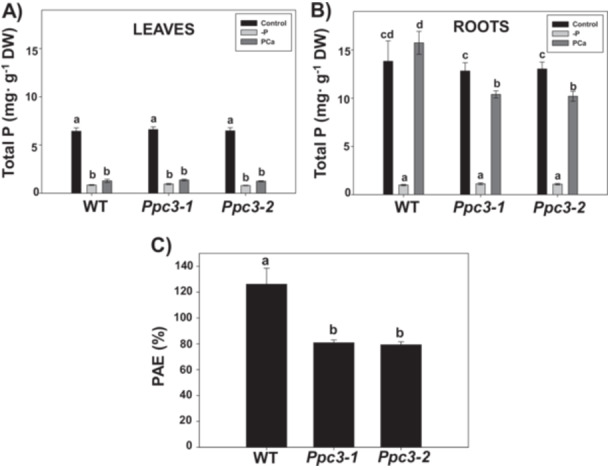
Total P accumulation and phosphate acquisition efficiency (PAE) in WT and *Ppc3* plants. Total P was measured in (A) leaves and (B) roots from WT and silenced lines, as described in Section 5. Bars indicate mean ± SE (*n* = 4–6). (C) Phosphate acquisition efficiency (PAE) was estimated as the percentage of P accumulated with PCa compared to P accumulated with soluble phosphate in roots, using data from panel (B). Columns that do not have a common letter are significantly different by Duncan's multiple range test (*p* < 0.05).

To analyze the success of PSR in PAE in WT and silenced plants, we measured total P accumulation in leaves and roots. As expected, the level of this element dropped drastically in all plants in ‐P treatments, both in leaves (Figure 9A) and in roots (Figure 9B). PCa treatment increased significantly the amount of P accumulated in roots from WT plants, reaching similar levels than in the presence of soluble phosphate (control conditions). Dissimilarly, in silenced *Ppc3* plants the level of P in roots with PCa was significantly lower than in control conditions (Figure 9B). Finally, we estimated the PAE, expressed as the percentage of P accumulated with PCa compared to P accumulated with soluble phosphate in roots (Narang et al. 2000) (Figure 9C). Silenced plants showed a ~30% reduction in PAE, reaching values of 113.8%, 81.0% and 78.2% for WT, *Ppc3*‐1 and *Ppc3*‐2 lines, respectively. In leaves, PCa treatment barely increased the level of P in all plants, indicating that P is not translocated to shoots when P is applied in the form of insoluble phosphate and that PCa imposes phosphate starvation. During long‐term Pi starvation, plants can accumulate higher amounts of Fe leading to toxicity (Misson et al. 2005). However, no toxic levels of Fe (Yamauchi 1989) or symptoms of iron toxicity were found in any plant or treatment. Fe was slightly accumulated only in *Ppc3‐1* leaves by PCa, and reduced by ‐P and PCa treatments in roots from all lines (Supporting Information S1: Figure S7). Finally, ATP levels were reduced by ‐P treatment in leaves and roots in all plants, whereas PCa recovered this level in leaves only in *Ppc3‐1* plants, but not in WT and *Ppc3‐2* plants or in roots from any line (Supporting Information S1: Figure S8). ATP level in silenced roots in control conditions was lower than the level in WT roots, indicating that silenced plants experience energy problems even in control conditions.

## 3 Discussion

Phosphate starvation is one of the major concerns for agriculture, with about 70% of arable soils affected by this stress. Although it may be present in the soil, its complexation with metal cations (e.g., Ca^2+^, Fe^3+^ or Al^3+^) makes phosphate insoluble and prevents its absorption by plants. Plants develop a plethora of biochemical and morphological responses to circumvent this problem, known as PSRs. Among them, PEPC has been described to be induced in roots from many plants in response to phosphate starvation (Plaxton and Tran 2011; Dissanayaka et al. 2021). However, most analysis on PEPC induction in response to phosphate starvation have been conducted in the absence or in the presence of very low amounts of soluble phosphate in the media. Thus, making not possible to assess the beneficial effect of PEPC induction, as there is no phosphate to be absorbed. Therefore, we conducted experiments, not only in the absence of phosphate (‐P experiments), but also in the presence of insoluble calcium phosphate (PCa experiments) where a successful PSR would rise the uptaking of P. In addition, we conducted the analysis using modified sorghum lines where the main PEPC isozyme from roots, SbPPC3, has been silenced (*Ppc3* lines) (de la Osa et al. 2022) to test the importance of this particular isozyme in PSR. Phosphate treatments induced PEPC activity, immune‐reactive PTPC polypeptides, and the expression of the non‐photosynthetic PEPCs Sb*PPC2* and Sb*PPC3* in roots from WT plants, with higher induction detected in ‐P compared to PCa. Silenced *Ppc3* plants did not show induction in PEPC activity, immune‐reactive PTPC polypeptides, or Sb*PPC3* expression, whereas Sb*PPC2* transcripts were detected similarly to WT plants, suggesting that SbPPC3 is the main PEPC isozyme responsible for the induction detected in sorghum roots. In leaves, the induction of Sb*PPC2* and Sb*PPC3* expression was not translated into a higher PEPC or immune‐reactive PTPC polypeptides, due to the presence of the photosynthetic PEPC isozyme (SbPPC1) (de la Osa et al. 2022).

The maximum quantum yield of photosystem II (Fv/Fm) is universally used as an indicator for plant stress (Maxwell and Johnson 2000). Optimal values of this parameter in the dark‐adapted plants are around 0.83 for most species, with lower values indicating that the plant has been exposed to stress and show photoinhibition. Anthocyanins are a class of red/purple‐coloured flavonoids that help to protect chloroplasts from photoinhibitory damage during Pi‐limited photosynthesis, and is commonly used as marker for phosphate stress (Dissanayaka et al. 2021). The reduction in Fv/Fm and the accumulation of anthocyanins by PCa was higher in silenced plants compared to WT plants, demonstrating that silenced plants are more stressed in the presence of insoluble phosphate in the media than WT plants.

Proteins containing the SPX (YG1/PHO81/XPR1) domain have been involved in responses to environmental cues or internal regulation of nutrition homeostasis. Rice plants contain 6 SPX proteins (OsSPX1‐6) with different patterns of expression and location. Among them, OsSPX1 is located in the nucleus, induced in Pi starved plants, and regulates the expression of other SPX proteins (Wang et al. 2009). The expression of SPX1 is also induced in other plants such as Arabidopsis or soybean under phosphate starvation (Duan et al. 2008; Zhang et al. 2016). The orthologue for SPX1 in sorghum, SbSPX1 (Sb10g185200), and the Pi‐induced phosphate transporter SbPHT1 (Sb01g046890), were used as molecular markers for PSR in sorghum roots (Walder et al. 2015). The expression of these markers was significantly higher in silenced roots compared to WT with PCa. Moreover, R/S ratio was similar in ‐P and PCa conditions in silenced, but not in WT plants. Altogether, these results show that, in *Ppc3* plants, PSR markers are more activated compared to WT plants in the presence of PCa, suggesting that WT plants perform better than silenced *Ppc3* plants in the presence of insoluble phosphate.

Apart from the induction of PEPC activity in WT roots, phosphate starvation altered other metabolic enzymes, mainly increasing CS and inhibiting ACO and ICDH activities. Overexpression of CS in Arabidopsis (Koyama et al. 2000), or MDH in cotton (Wang et al. 2015), have proven to improve growth under phosphate starvation conditions. In a proteomic analysis, maize plants showed higher CS and MDH and lower ACO and ICDH activities in roots, accompanied by an increase in citrate accumulation (Alexova and Millar 2013). Although we found a similar effect of phosphate treatments on TCA enzymes in sorghum roots, except for MDH that was unaffected, citrate or malate were not accumulated in this tissue compared to control conditions. Succinate levels were reduced in roots in response to phosphate treatments (‐P and PCa), with lower amounts in silenced plants compared to WT, whereas the amino acids glutamate and aspartate were accumulated. The accumulation of amino acids and reduction of OAs have been previously showed in barley roots under severe P deficiency (Huang et al. 2008). It is worth to note that, among all enzymes analyzed, PEPC is the metabolic enzyme activated in a higher degree in response to phosphate starvation, by twofold compared to control conditions. This could explain, at least partially, the reduction in PEP levels found in roots from WT plants under phosphate treatments (‐P and PCa). Surprisingly, silenced plants showed lower PEP levels in roots compared to WT plants in control conditions, and similar levels under phosphate treatments. However, PEP cannot be seen only as substrate for PEPC, but also as the origin of other three major metabolic routes: mitochondrial respiration associated with PK, the shikimate‐phenylpropanoid pathway for phenolic compounds production, and the MEP pathway for isoprenoid formation (Dizengremel et al. 2012). Taking into account that PK is not induced in silenced plants, the shikimate and MEP pathways could account for this reduction in PEP levels in roots from silenced plants in control conditions. In addition, as Sb*PPC2* is similarly induced by phosphate treatments in WT and silenced lines, part of PEP may also be used by this PEPC isozyme in *Ppc3* plants under phosphate starvation. The induction of CS and the inhibition of ACO in P starved plants prevents the synthesis of isocitrate and leads to the accumulation of citrate to be exudated from roots for phosphate solubilisation in the rhizosphere (Plaxton and Tran 2011; Gupta et al. 2012). When analyzing root exudates, citrate was the sole OA which secretion was clearly induced in response to phosphate treatments. Surprisingly, silenced plants secreted higher amounts of citrate under any phosphate treatment than WT plants, indicating that PPC3 activity might not be essential for citrate exudation in roots, with the induction of CS and the inhibition of ACO and ICDH probably accounting for this exudation increase. In addition, the other PEPC isozyme induced in response to phosphate starvation, SbPPC2, could be participating in the synthesis and exudation of citrate. In previous results, PPC3 was not important for the exudation of citrate after 2 days of aluminum treatment, but it was for its exudation after 1 week of cadmium stress (Pérez‐López et al. 2023b), indicating that the implication of this isozyme in OA exudation changes with time. Moreover, PTMs in metabolic enzymes could modify the enzymatic activity in vivo, thus increasing citrate synthesis and exudation without changes in the amount of proteins (O'Leary and Plaxton 2020). In a recent study, phosphate starvation changed the abundance of ~1100 proteins and the phosphorylation state of more than 500 in Arabidopsis cell cultures (Mehta et al. 2021), indicating that, in addition to changes in protein quantity, changes in PTMs are of outmost importance for PSR. Nevertheless, the exudation of other OAs, particularly succinate, which accumulation in roots was also lower in silenced plants, was smaller in *PPC3* lines compared to WT, both in control and in PCa conditions, although their exudation was not induced by phosphate treatments. In a study on several Arabidopsis accessions with different PAE, citrate and malate were the OAs more exudated in the presence of insoluble phosphate or without phosphate. Notwithstanding, succinate, 2‐OG and fumarate have also been reported to be able to mobilise P, although to a lower extent than citrate or malate (Kpomblekou‐A and Tabatabai 1994, 2003). In rice plants expressing a photosynthetic PEPC from maize, oxalate but not citrate exudation was induced in response to low levels of soluble phosphate (Begum et al. 2005). In the monocot *Brachypodium distachyon*, the most exudated OA in control conditions was citrate, followed by malate, and succinate, glutamate and aspartate in similar amounts (Kawasaki et al. 2016). Therefore, different plants can exudate different OAs under the same conditions (Schöttelndreier. 2001). In our conditions, the most exudated OA in WT plants in control conditions was glutamate, followed by succinate and pyruvate, aspartate, malate, and, finally, citrate. Interestingly, modified *Ppc3* plants exudate lower amounts of these OAs, although their exudation was not induced by phosphate starvation. Although citrate was the sole OA which exudation was induced in ‐P in WT plants, the amount of citrate was similar to the amount of glutamate or pyruvate, and particularly lower than the amount of succinate. In addition, the exudation of succinate is reduced by twofold in *Ppc3* plants compared to WT in all conditions analyzed. Finally, when analyzing the levels of P accumulated in roots, silenced plants showed lower accumulation of this element in the presence of calcium phosphate, leading to ~30% reduction in PAE in *Ppc3* compared to WT plants. Therefore, although citrate and malate exudation is not reduced in ‐P or PCa conditions in silenced plants, the lower exudation of other OAs, such as succinate, could affect, at least partially, to the P solubilisation capacity of *Ppc3* plants, leading to a lower PAE and accumulation of this element in roots, and to the phenotype observed.

PEPC induction is not the sole response to phosphate starvation. Plants develop multiple anatomical, physiological, biochemical and molecular responses to improve phosphate uptake when this nutrient is scarce or not available. Hence, its logical that, when one of these responses fails, e.g. PEPC induction, other responses can compensate, at least partially, to keep taking up P. This seems to be the case in *Ppc3* lines, where plants maintain activated other starvation responses when phosphate is insoluble, whereas WT do not. Albeit WT and silenced lines showed a similar phenotype when growing in the absence of phosphate (‐P), this was not the case in the presence of insoluble PCa, where silenced lines showed to be more stressed compared to WT. This stressed phenotype, and the lower PAE found in silenced *Ppc3* plants growing with calcium phosphate, supports the importance of PPC3 as a part of PSR and the significance of PEPC induction under phosphate deficiency.

## 4 Conclusion

Phosphate starvation is a major problem for agriculture, with reserves of rock phosphate used as fertilisers scarce and expected to be exhausted by the next 50–100 years. Thus, it is of outmost importance to develop innovative approaches to decrease the need for Pi fertilisers. One of these approaches could be achieved through manipulating crops to enhance their uptake of Pi from the soil, i.e. increased PAE (Dissanayaka et al. 2021). In this manuscript, we demonstrate the importance of PEPC induction in roots in PSR, and PPC3 as the main PEPC isozyme responsible of such induction. The stressed phenotype observed in silenced, but not in WT plants, and the reduction in PAE in the presence of PCa, points to this particular PEPC isozyme as a target to improve PAE in sorghum and other crops.

## 5 Materials and Methods

### 5.1 Plant Material and Growth Conditions

Sorghum (*Sorghum bicolor* L.) WT plants used in this study correspond to the public genotype P898012. *Ppc3* plants were generated by transforming sorghum immature embryos genotype P898012 with *Agrobacterium tumefaciens* bacteria harbouring the binary vector pFGC161 containing a specific 300 bp fragment of Sb*PPC3* gene (bases from 1260 to 1560 in the CDS) in the sense and anti‐sense orientations. *Ppc3‐1* and *Ppc3‐2* are independent transgenic lines showing approximately a 90% inhibition Sb*PPC3* expression, both in leaves and roots (de la Osa et al. 2022). Seeds were surface sterilised with 50% (v/v) bleach and 0.1% Triton X‐100 for 30 min and rinsed 8–10 times with sterile water. Seeds were placed in moist sterile filter papers for 3 days in darkness at 25°C. Seedlings were transferred to 1 L polyethylene pots filled with nitrate‐type nutrient solution (Hewitt 1966) and grown in 12 h light/dark cycles (25°C, 60% relative humidity and 20°C, 70% relative humidity for each photoperiod, respectively), 350 μmol m^−2^ s^−1^ PAR light intensity.

Phosphate treatments were applied to plants 1 week after transferred to the hydroponical cultures. P was added as 1.2 mM NaH_2_PO_4_, for control or soluble phosphate treatments (C), as 0.6 mM Ca_3_(PO_4_)_2_, for insoluble phosphate treatments (PCa), or omitted for phosphate starvation treatments (‐P). Media was renewed weekly. The solubility product constant (K_sp_) of Ca_3_(PO_4_)_2_ is 2.07 × 10^−33^ (mol^3^ L^−3^), what means phosphate available in PCa treatment to be around 0.107 μM. The initial pH was 6.8 for C and ‐P, and 7.8 for PCa treatments. Twenty‐four hours after replacing the media, pH was 6.8 in all plants and treatments, and it remained approximately constant until renewed 6 days later, indicating that silencing of Sb*PPC3* does not affect the acidification of the media (data not shown). Three weeks after applying phosphate treatments, 2–3 fully expanded young leaves from each plant and total roots were collected, grinded with a mortar and pestle with liquid nitrogen and stored at −80°C until use. Each biological replicate was composed of tissue from three individual plants in the same pot.

The root/shoot (R/S) ratio was calculated based on the fresh weight of roots and shoots. Roots were harvested, washed with water, dried in absorbent paper and weighted. For shoots, the total aerial part was harvested and weighted. Each biological replicate was composed of three individual plants in the same pot.

### 5.2 Enzyme Extraction and Analysis

Protein extracts for PEPC activity, SDS‐PAGE electrophoresis, and gel blots analysis, were obtained by grinding 0.2 g or 0.5 g fresh weight from leaves or roots, respectively, in 1 mL of extraction buffer containing: 0.1 M Tris‐HCl pH 7.5, 5% (v/v) glycerol, 1 mM EDTA, 10 mM MgCl_2_, 1 mM phenylmethylsulfonyl fluoride, 10 μg mL^−1^ chymostatin, 10 μM leupeptin, and 14 mM β‐mercaptoethanol. The homogenate was centrifuged at 17 000*g* for 5 min at 4°C, and the supernatant was used for determination of enzymatic activities.

PEPC activity was measured spectrophotometrically at pH 8 and 2.5 mM PEP as described in Echevarria et al. (1994). A single enzyme unit (U) is defined as the amount of PEPC that catalysers the carboxylation of 1 μmol of PEP per minute at pH 8, 30°C.

For the rest of enzymatic activities, aliquots of 100 mg of frozen leaf and root powder were extracted in 1 mL of extraction buffer as described in Vega‐Mas et al. (2019). Enzyme activities were determined by spectrophotometric methods in a 96‐well microplate reader. Assays to measure CS, isocitrate dehydrogenase (ICDH), malic dehydrogenase (MDH), NAD‐malic enzyme (NAD‐ME), and NADP‐malic enzyme (NADP‐ME) were performed following Vega‐Mas et al. (2019), and pyruvate kinase (PK) as described in Gibon et al. (2004). Aconitase was measured as described by de Vos et al. (1986).

### 5.3 Electrophoresis and Gel Blot Analysis

Protein samples were denatured by heating in the presence of loading buffer (100 mM Tris‐HCl, pH 8, 25% [v/v] glycerol, 1% [w/v] SDS, 10% [v/v] β‐mercaptoethanol, and 0.05% [w/v] bromophenol blue). Denatured proteins (10 µg for leaves extracts and 50 µg for roots extracts) were separated by SDS‐PAGE in a Mini‐Protean® III‐2D Cell (Bio‐Rad) and electroblotted onto a nitrocellulose membrane in a semidry transfer blot system (Bio‐Rad Laboratories). Polyclonal antibodies against native C_4_‐photosynthetic PEPC from sorghum leaves (anti‐C_4_ PTPC) were prepared as described in Pacquit et al. (1995). These antibodies recognise both photosynthetic and non‐photosynthetic PEPCs (Ruiz‐Ballesta et al. 2016; Arias‐Baldrich et al. 2017). Anti‐tubulin antibodies were used as loading control. Immunolabeled proteins were detected as described in Arias‐Baldrich et al. (2017). For ratio PEPC/tubulin calculation, band intensities were measured using Image Studio TM Lite software (LI‐COR Biosciences).

### 5.4 Protein Quantification

Protein concentrations were determined using the method of Bradford (1976) with BSA as the standard.

### 5.5 Gas Exchange Measurements

Net photosynthetic rate (A) and stomatal conductance (g_s_) were determined in the youngest fully developed leaf at 350 μmol m^−2^ s^−1^ PAR, a CO_2_ concentration of 400 ppm, and ambient relative humidity, using a portable photosynthesis analyzer LI‐6400XT (LI‐COR).

### 5.6 Maximum Quantum Yield of PSII (F_v_/F_m_) Measurement

Maximum quantum yield of PSII (F_v_/F_m_) was measured after 3 weeks of treatment in the youngest fully developed leaf from dark adapted plants (10 h darkness, 2 h before dawn), using a portable modulated fluorimeter FMS‐2 (Hansatech Instrument Ltd., England). The minimal fluorescence level in the dark‐adapted state (F_0_) was measured using a modulated pulse (< 0.05 µmol m^−2^ s^−1^ for 1.8 µs). The data stored were an average taken over a 1.6 s period. Maximal fluorescence in this state (F_m_) was measured after applying a saturating actinic light pulse of 15 000 µmol m^−2^ s^−1^ for 0.7 s. Values of the variable fluorescence were calculated from F_0_ and F_m_ (F_v_ = F_m_ − F_0_). F_v_/F_m_ (ratio of variable to maximal fluorescence) correlates with the number of functional PSII reaction centres and dark adapted and can be used to quantify photoinhibition (Krivosheeva et al. 1996).

### 5.7 Photosynthetic Pigments

Chlorophylls (a and b) and carotenoids were extracted from leaves with 80% acetone, as described in Arias‐Baldrich et al. (2015).

### 5.8 Anthocyanins Quantification

Anthocyanins were measured in leaves as described by Mancinelli (1984) with minor modifications. Briefly, 0.15 g leaves tissue were grinded in liquid nitrogen, and anthocyanins extracted in 5 mL acidified (1% HCl; v/v) 80% ethanol (v/v) for 24 h at 4°C in a circular stirrer. Samples were centrifuged at 12 000 rpm for 5 min at 4°C, and the supernatant used for anthocyanins quantification. Absorbance was measured at 530 and 657 nm, and the formula A_530_ − 0.33A_657_ used to compensate for the chlorophyll absorbance at 530 nm.

### 5.9 ATP Quantification

ATP was quantified in leaves and roots using a luciferin‐luciferase ATP bioluminescent assay kit (Sigma) in a Luminoskan TL luminometer (LabSystem Oy, Helsinki, Finland), as described in García‐Mauriño et al. (2005).

### 5.10 RNA Extraction and cDNA Synthesis

Total RNA was extracted from 100 mg of frozen, powdered leaves or roots using the RNeasy Plant Mini kit (Qiagen). Extracted nucleic acids were DNAase treated to wipe out genomic DNA. RNA concentration was determined using Nanodrop 2000 (Thermo). Reverse‐transcription reactions were performed using 1 μg of purified total RNA and the Transcriptor First Strand cDNA Synthesis Kit (Roche), following the manufacturer's instructions. cDNA synthetised was used for qPCR and microarray experiments.

### 5.11 Quantitative Real‐Time PCR (qPCR)

Quantitative PCR reactions (qPCR) were performed in a final volume of 20 μL consisting of 1 μL of the cDNA, 15 μM of the specific primers (Supporting Information S2: Table S1), and 10 μL of SensiFAST SYBR No‐ROX kit (Bioline). PCR was conducted on the Light Cycler 480 II Real‐Time PCR System (Roche), and the threshold cycles (Ct) were determined using the Light Cycler 480 software for all treatments. To normalise the obtained values, actin was used as internal control in each sample. Relative expression data were obtained through Livak method (2^−ΔΔCt^; Livak and Schmittgen 2001), assigning the value 1 for Sb*PPC3* expression in WT leaves in control conditions.

### 5.12 Root Exudation Collection and Analysis of OAs in Exudates by UHPLC‐HRMS/MS

Three weeks after phosphate treatments, roots from three independent plants in the same hydroponic were washed with distilled water and submerged in bakers with 250 mL mili‐Q water for 3 h to collect exudates. A total of 50 mL of collected exudates were lyophilised, resuspended in 1 mL of mili‐Q water, and filtered in 0.2 µm nylon filters. OAs in root exudates were analyzed by ultrahigh‐performance liquid chromatography coupled with high‐resolution tandem mass spectrometry (UHPLC‐HRMS/MS) at the Mass Spectrometry Services (CITIUS, University of Sevilla). UHPLC was carried out using an analytic column Acquity UPLC HSS T3 (2.1 × 100 mm) 1.8 µm particle size with a binary pump Dionex Ultimate RS to inject the samples. The analysis conditions were set at a flow rate of 0.45 mL/min and an injection volume of 5 μL. OAs were eluted using a gradient of buffer A (0.1% formic acid) and buffer B (0.1% formic acid in ACN). OAs eluted were analyzed using a quadrupole Orbitrap QExactative mass spectrometer (ThermoFisher Scientific, USA) equipped with an electrospray (HESI‐II) operating at −2.5 kV. Results were analyzed using the Tracefinder software (Thermo Scientific, USA). For OA quantification, internal standards at different concentrations were used to obtain calibration curves. Each biological replicate was composed of the exudates produced by three plants in the same hydroponic. Each sample was analyzed twice (technical replicates).

### 5.13 OA Quantification in Roots

After 3 weeks with phosphate treatments, root tissue was harvested, grinded in liquid N_2_ and lyophilised. A total of 200 mg of lyophilised root tissue were homogenised in 1.8 mL of methanol:water:chloroform (4:4:10). After centrifugation, the upper layer was recovered, vacuum dried and the pellet resuspended in 1 mL of ultrapure water. Extracts were filtered through a 0.22‐μm PES filter and OA quantification was performed by ion chromatography (Dionex ICS‐5000, Thermo Scientific) at the Advanced Research Facilities (SGIker) of the UPV/EHU. For anion separation, a Dionex Ion Pac AS11‐HC‐4 μm analytical column was employed, preceded by a Dionex Ion Pac AG 11‐HC‐4 μm guard column. The eluent used was supplied by a Dionex EGC 500 KOH Eluent Generator Cartridge, complemented with a continuously regenerated Dionex CR‐ATC 500 Anion Trap column, utilising a KOH gradient up to 60 mM. The analysis conditions were set at a flow rate of 0.4 mL/min and an injection volume of 6.5 μL. Detection was carried out via suppressed conductivity with a Dionex AERS 500 2 mm, set at 60 mA in external water mode. Each analysis had a duration of 48 min.

Identification and quantification of metabolites was carried out by the use of standards to create a standard curve, and analysed by the Automated Mass Spectral Deconvolution and Identification System (AMDIS) software (http://chemdata.nist.gov/mass-spc/amdis/downloads). Quantification of metabolites was carried out using the Qual Browser of the Xcalibur data system (Thermo).

### 5.14 Total P and Fe Measurement

For total P and Fe quantification, leaves and roots samples were dried in an oven at 60°C for 7 days, and grinded with a mortar and a pestle. A total of 0.25 g of dried powder were burned to ashes at a temperature of 550°C for 8 h using a muffle furnace. A total of 12 mL 1 N HCl were added to ashes and incubated in a heating plate for 15 min for acidic digestion. Total P was measured spectrophotometrically by the ammonium molybdate and potassium antimonyl tartrate in acidic medium method, as described by Murphy and Riley (1962). Fe was quantified by atomic absorption using an iCE 3500 spectrometer (Thermo Scientific). P and Fe were quantified at the Agronomic Research Services (CITIUS, University of Seville).

### 5.15 Statistical Analysis

Data were analyzed by ANOVA and means compared by Duncan's Multiple Range Test, as indicated in figure legends. A *p* value of < 0.05 was considered to be statistically significant. All analyzes were conducted using SPSS statistic 25 (IBM).

## Conflicts of Interest

The authors declare no conflicts of interest.

## Supporting information


**Supplemental Fig. S1:** Effect of phosphate starvation in sorghum leaves from WT plants. **Supplemental Fig. S2:** Effect of phosphate starvation in sorghum leaves from WT and Ppc3 plants. **Supplemental Fig. S3:** Effect of phosphate starvation in photosynthetic pigments of WT and Ppc3 plants. **Supplemental Fig. S4:** Effect of phosphate starvation in growth of WT and Ppc3 plants. **Supplemental Fig. S5:** Effect of phosphate starvation on TCA enzymes in roots from WT and Ppc3 plants. **Supplemental Fig. S6:** Effect of phosphate starvation on TCA enzymes in leaves from WT and Ppc3 plants. **Supplemental Fig. S7:** Total Fe accumulation in WT and Ppc3 plants. **Supplemental Fig. S8:** Effect of phosphate starvation on ATP levels in leaves and roots from WT and Ppc3 plants.

**Supplemental table S1:** List of primers used in this work. Nucleotides in red spam an exon‐exon junction.

## Data Availability

The data that support the findings of this study are available from the corresponding author upon reasonable request.
