# Supplementary material for: Unravelling the Significance of Phosphoenolpyruvate Carboxylase in Phosphate Starvation Responses
Source: Plant Cell Environ. 2025 Sep 24;49(1):177–92. doi: 10.1111/pce.70204 (PMC12675990; doi:10.1111/pce.70204)
Supplement: Supplementary file 1 — Supplemental Fig. S1: Effect of phosphate starvation in sorghum leaves from WT plants. Supplemental Fig. S2: Effect of phosphate starvation in sorghum leaves from WT and Ppc3 plants. Supplemental Fig. S3: Effect of phosphate starvation in photosynthetic pigments of WT and Ppc3 plants. Supplemental Fig. S4: Effect of phosphate starvation in growth of WT and Ppc3 plants. Supplemental Fig. S5: Effect of phosphate starvation on TCA enzymes in roots from WT and Ppc3 plants. Supplemental Fig. S6: Effect of phosphate starvation on TCA enzymes in leaves from WT and Ppc3 plants. Supplemental Fig. S7: Total Fe accumulation in WT and Ppc3 plants. Supplemental Fig. S8: Effect of phosphate starvation on ATP levels in leaves and roots from WT and Ppc3 plants. [file PCE-49-177-s002.pdf]

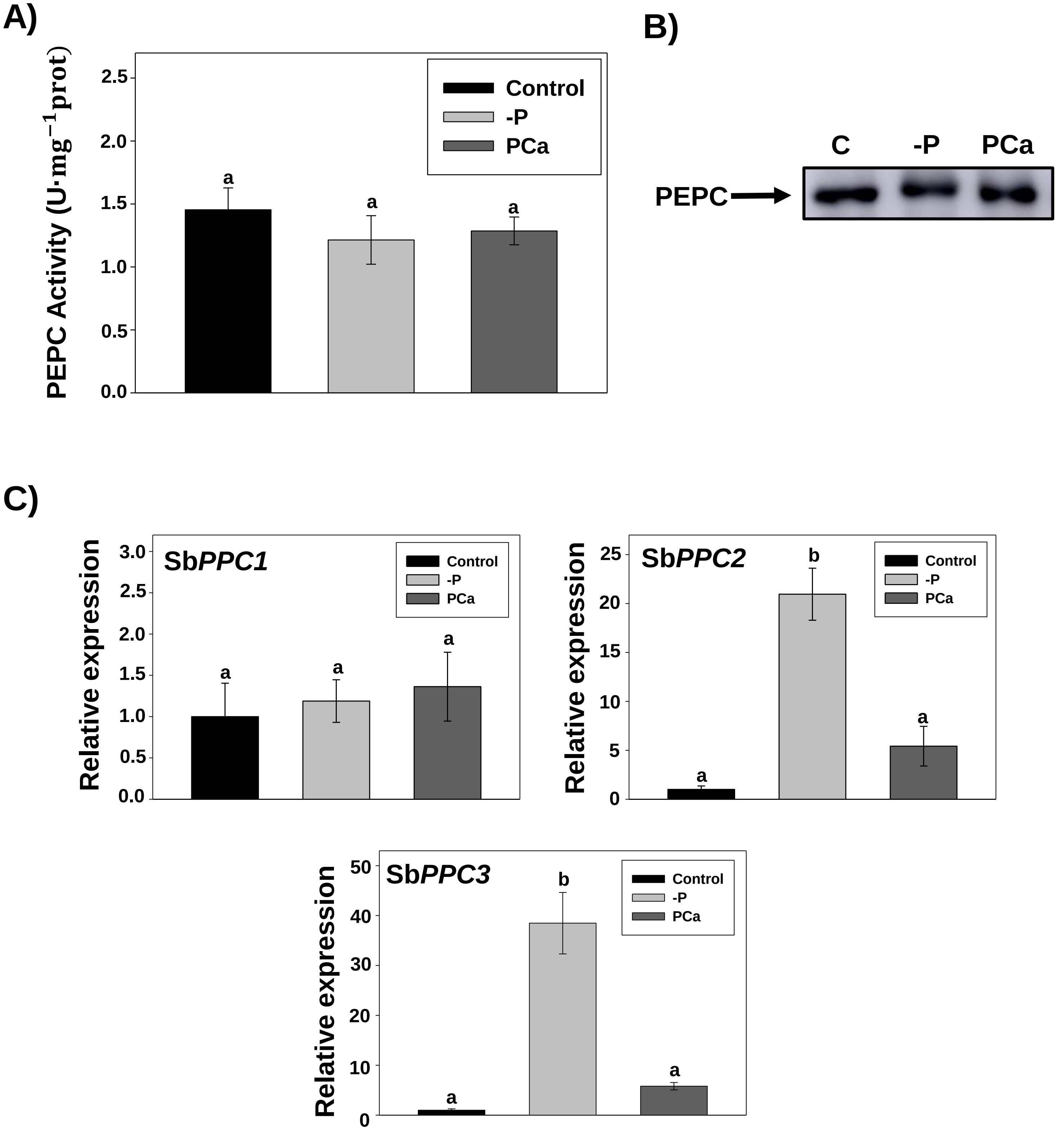

Supplemental Fig. S1. Effect of phosphate starvation in sorghum leaves from WT plants. Plants were grown hydroponically with nitrate-type nutrient solution (Hewitt) with 1.2 mM NaH_2PO_4 (soluble phosphate, Control), 0.6 mM $Ca_3(PO_4)_2$ (insoluble phosphate, PCa), or without phosphate (-P), for 3 weeks, as described in Materials and Methods. **(A)** PEPC activity in sorghum leaves. **(B)** Immunodetection of PEPC protein in sorghum leaf extracts. For analysis of protein levels, 10 µg per lane of total proteins were loaded for SDS-PAGE and transferred onto nitrocellulose membranes for blotting using anti-PTPC antibodies, as described in Materials and Methods. **(C)** Relative transcript abundance of Sb*PPC1*, Sb*PPC2* and Sb*PPC3* in sorghum leaves. For each gene, data are normalized to the transcript abundance in control conditions. Bars in (A) and (C) indicate mean ± SE (n = 4), where each biological sample is composed of a pool of leaves from three individual plants growing in the same pot. Columns that do not have a common letter are significantly different by Duncan's multiple range test ($P < 0.05$).

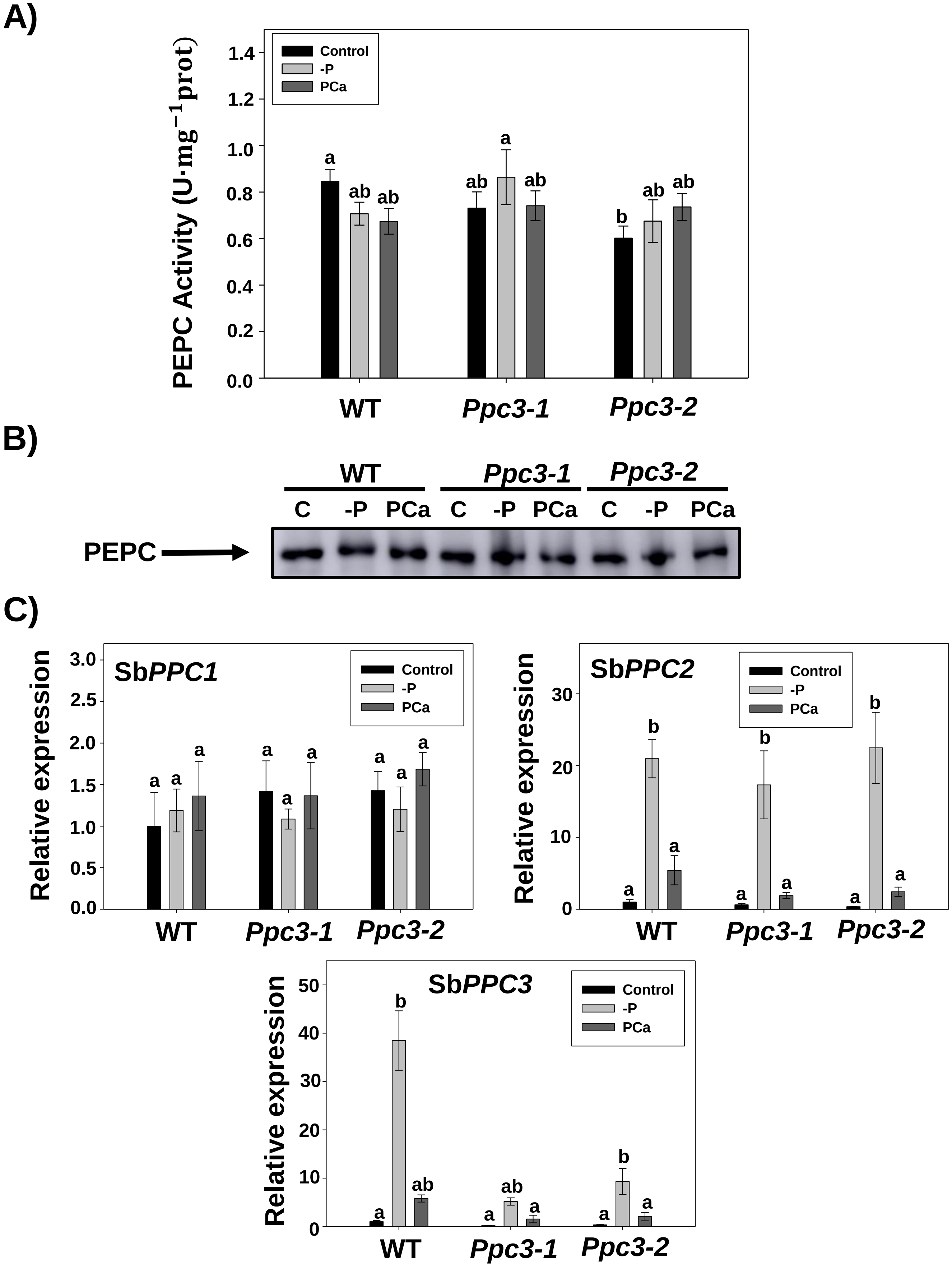

Supplemental Fig. S2. Effect of phosphate starvation in sorghum leaves from WT and *Ppc3* plants. Plants were grown hydroponically with nitrate-type nutrient solution with soluble phosphate (C), without phosphate (-P) or with calcium phosphate (PCa), as described in Fig. S1 and Materials and Methods. **(A)** PEPC activity in sorghum leaves. **(B)** Immunodetection of PEPC protein in sorghum leaf extracts. For analysis of protein levels, 10 µg per lane of total proteins were loaded for SDS-PAGE and transferred onto nitrocellulose membranes for blotting using anti-PTPC antibodies, as described in Materials and Methods. **(C)** Relative transcript abundance of Sb*PPC1*, Sb*PPC2* and Sb*PPC3* in sorghum leaves. For each gene, data are normalized to the transcript abundance in control conditions. Bars in (A) and (C) indicate mean ± SE (n = 4), where each biological sample is composed of a pool of leaves from three individual plants growing in the same pot. Columns that do not have a common letter are significantly different by Duncan's multiple range test ($P < 0.05$).

A)

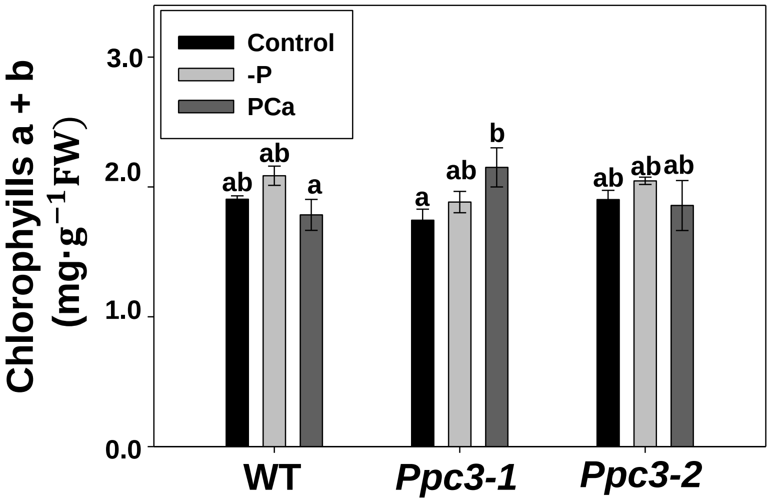

B)

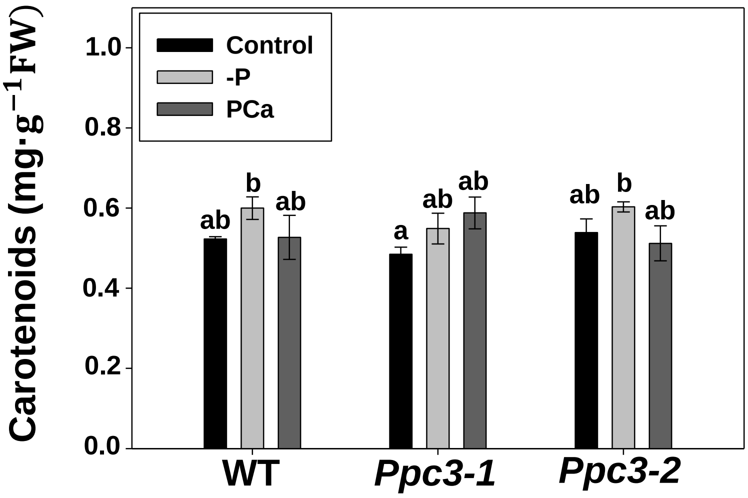

Supplemental Fig. S3. Effect of phosphate starvation in photosynthetic pigments of WT and *Ppc3* plants. Plants were grown hydroponically with nitrate-type nutrient solution with soluble phosphate (C), without phosphate (-P) or with calcium phosphate (PCa), for 3 weeks, as described in Fig. S1 and Materials and Methods. **(A)** Total chlorophylls (a + b) **(B)** Carotenoids. Bars indicate mean ± SE (n = 4), where each biological sample is composed of fully developed young leaves from three individual plants growing in the same pot. Columns that do not have a common letter are significantly different by Duncan's multiple range test ($P < 0.05$).

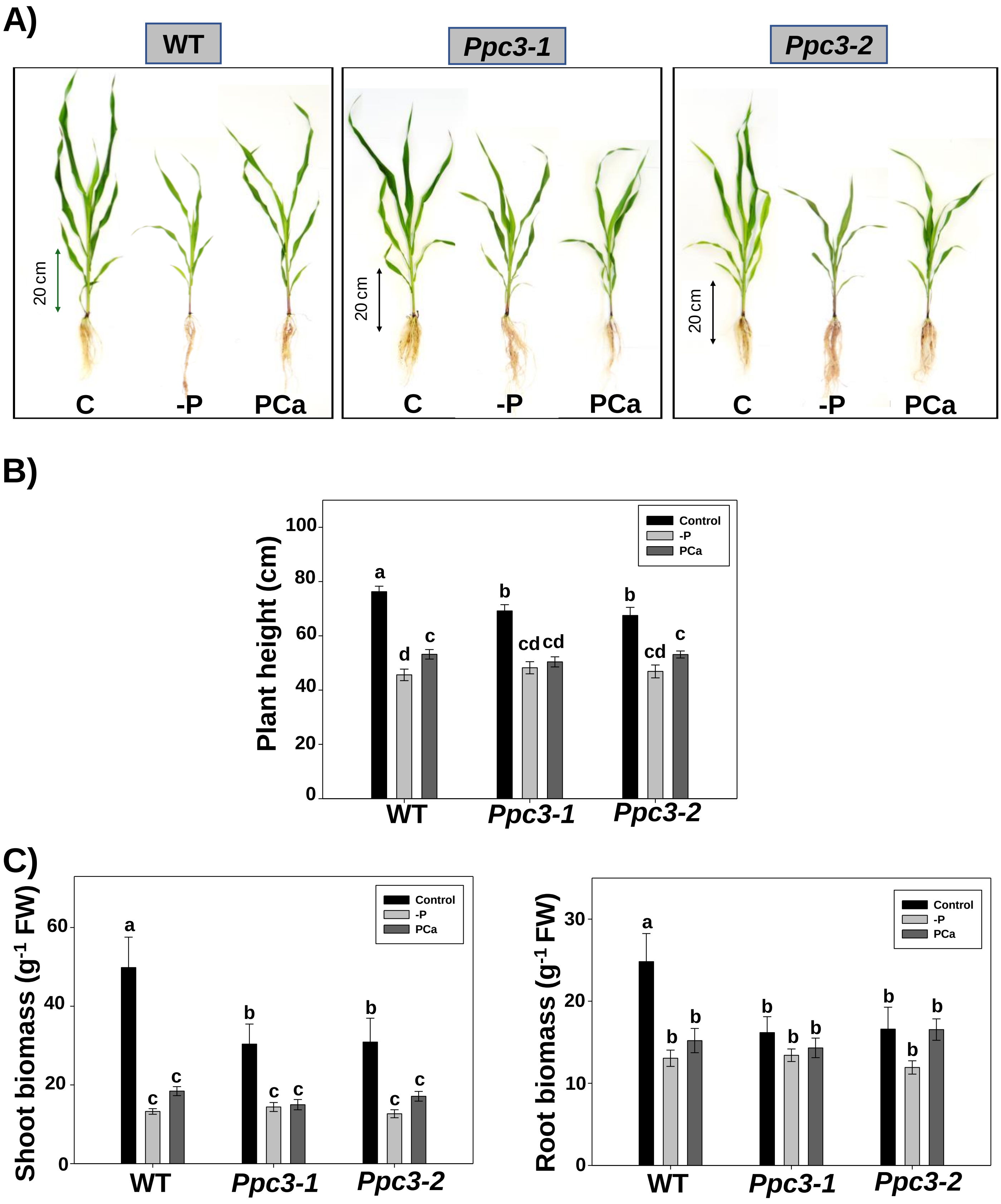

Supplemental Fig. S4. Effect of phosphate starvation in growth of WT and *Ppc3* plants. Plants were grown hydroponically with nitrate-type nutrient solution with soluble phosphate (C), without phosphate (-P) or with calcium phosphate (PCa), for 3 weeks, as described in Fig. S1 and Materials and Methods. **(A)** Representative image at the end of the treatments **(B)** Plant height (cm). Bars indicate mean ± SE (n = 27), where each data correspond to one individual plant **(C)** Fresh weight from shoots (left) and roots (right). Bars indicate mean ± SE (n = 7), where each biological sample is composed of three individual plants growing in the same pot. Columns that do not have a common letter are significantly different by Duncan's multiple range test ($P < 0.05$).

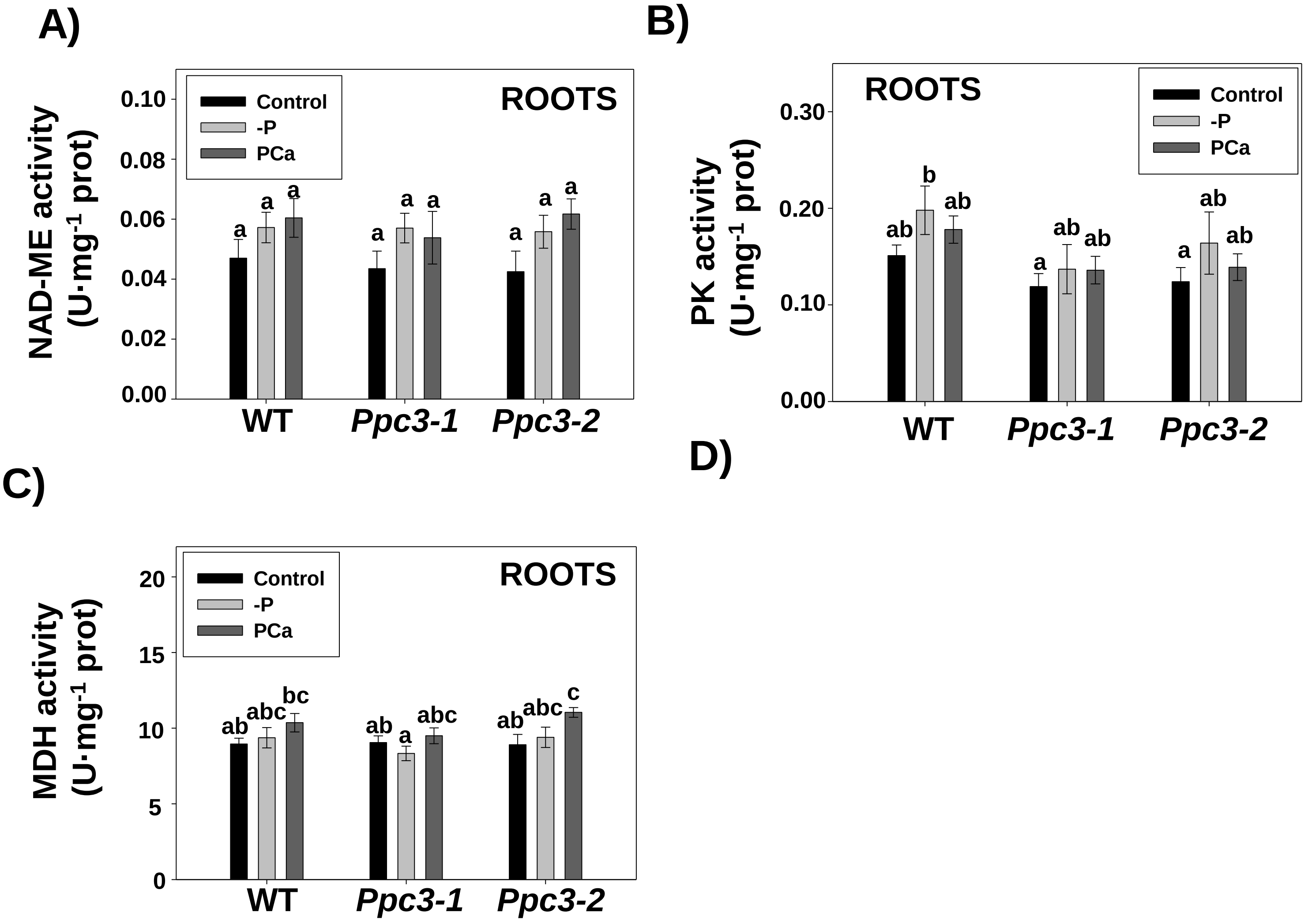

Supplemental Fig. S5. Effect of phosphate starvation on TCA enzymes in roots from WT and *Ppc3* plants. 3 weeks after growing hydroponically under phosphate treatments, plants were harvested and TCA enzymatic activities from roots analyzed as described in Materials and Methods section **(A)** NAD-malic enzyme (NAD-ME) **(B)** Pyruvate kinase (PK) **(C)** Malate dehydrogenase (MDH). Bars indicate mean ± SE (n =3-7). Columns that do not have a common letter are significantly different by Duncan's multiple range test (P < 0.05).

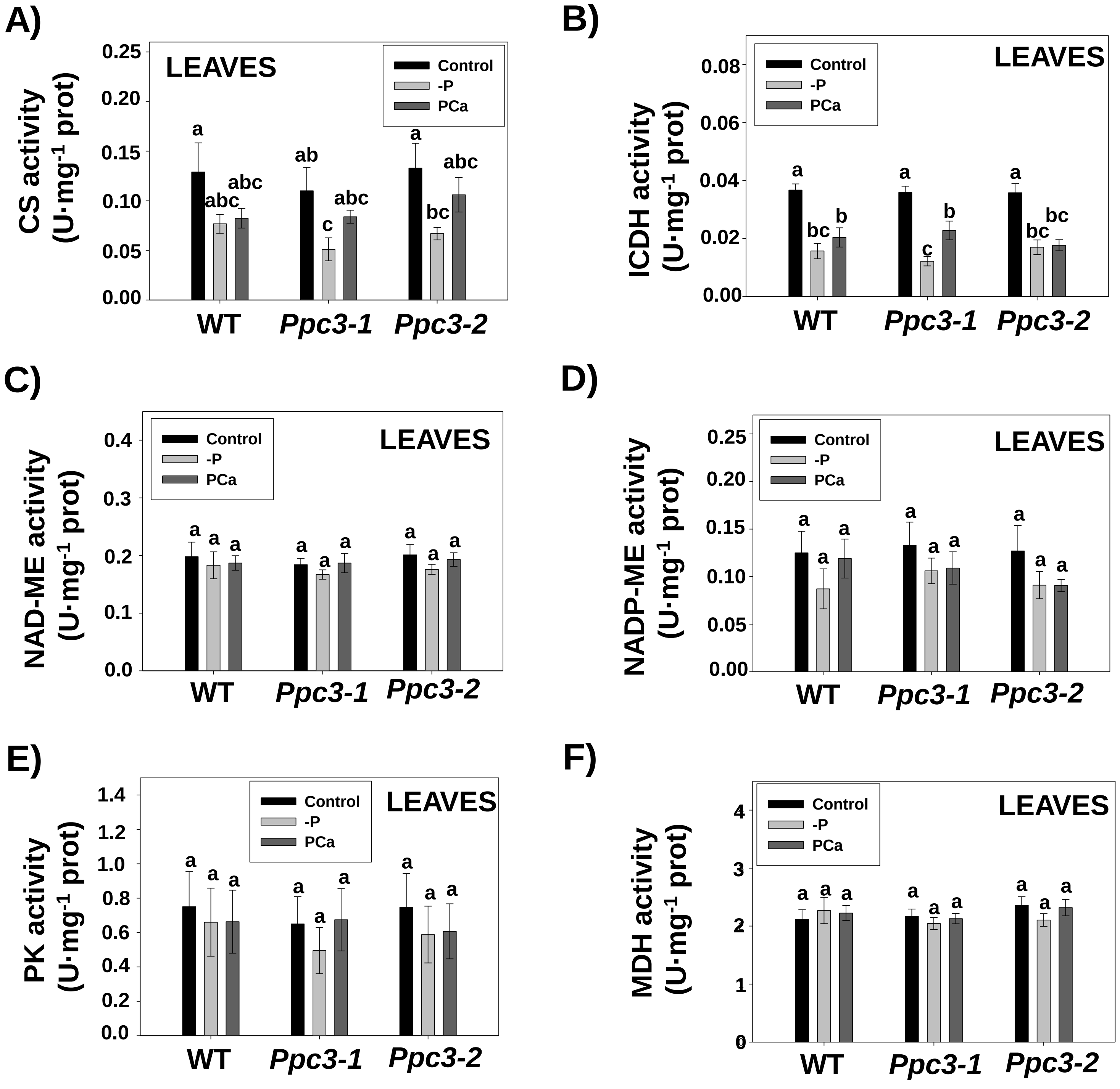

Supplemental Fig. S6. Effect of phosphate starvation on TCA enzymes in leaves from WT and *Ppc3* plants. 3 weeks after growing hydroponically under phosphate treatments, plants were harvested and TCA enzymatic activities from leaves analyzed, as described in Materials and Methods **(A)** Citrate synthase (CS) **(B)** Isocitrate deshydrogenase (ICDH) **(C)** NAD-malic enzyme (NAD-ME) **(D)** NADP-Malic enzyme (NADP-ME) **(E)** Pyruvate kinase (PK) **(F)** Malic deshydrogenase (MDH). Bars indicate mean ± SE (n =3-7). Columns that do not have a common letter are significantly different by Duncan's multiple range test ($P < 0.05$).

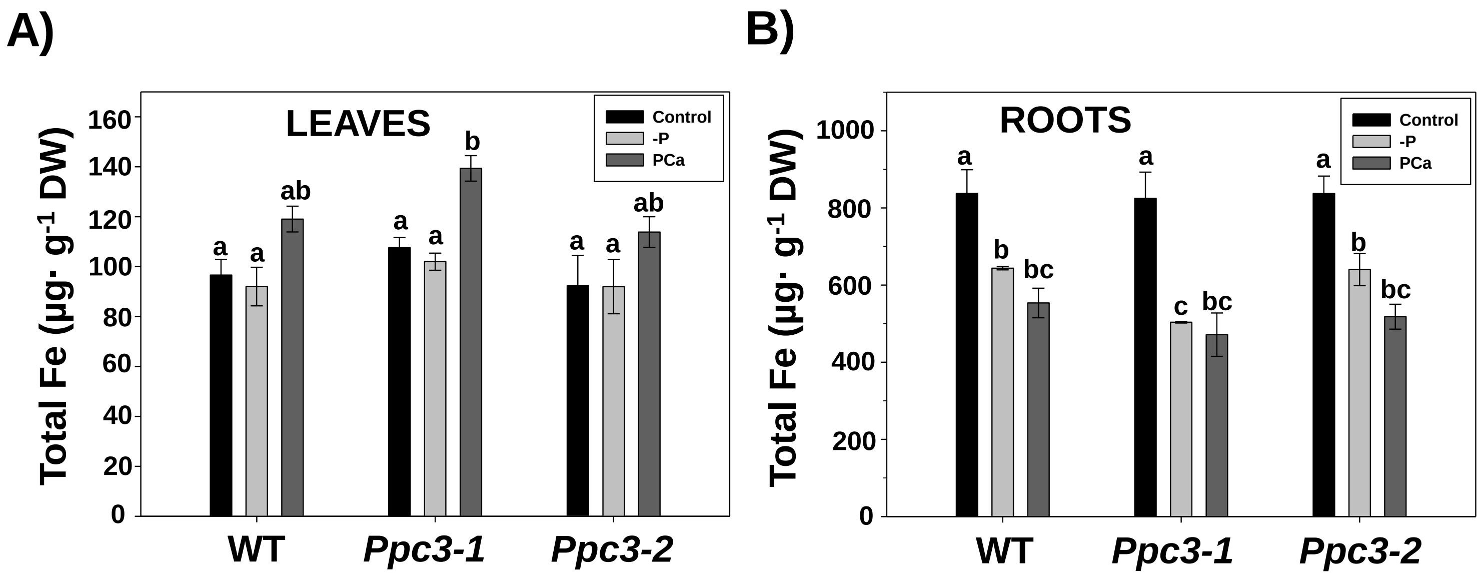

Supplemental Fig. S7. Total Fe accumulation in WT and *Ppc3* plants . Total Fe was measured in **(A)** leaves and **(B)** roots from WT and silenced lines, as described in Materials and Methods section. Bars indicate mean ± SE (n =3-5). Columns that do not have a common letter are significantly different by Duncan's multiple range test ($P < 0.05$).

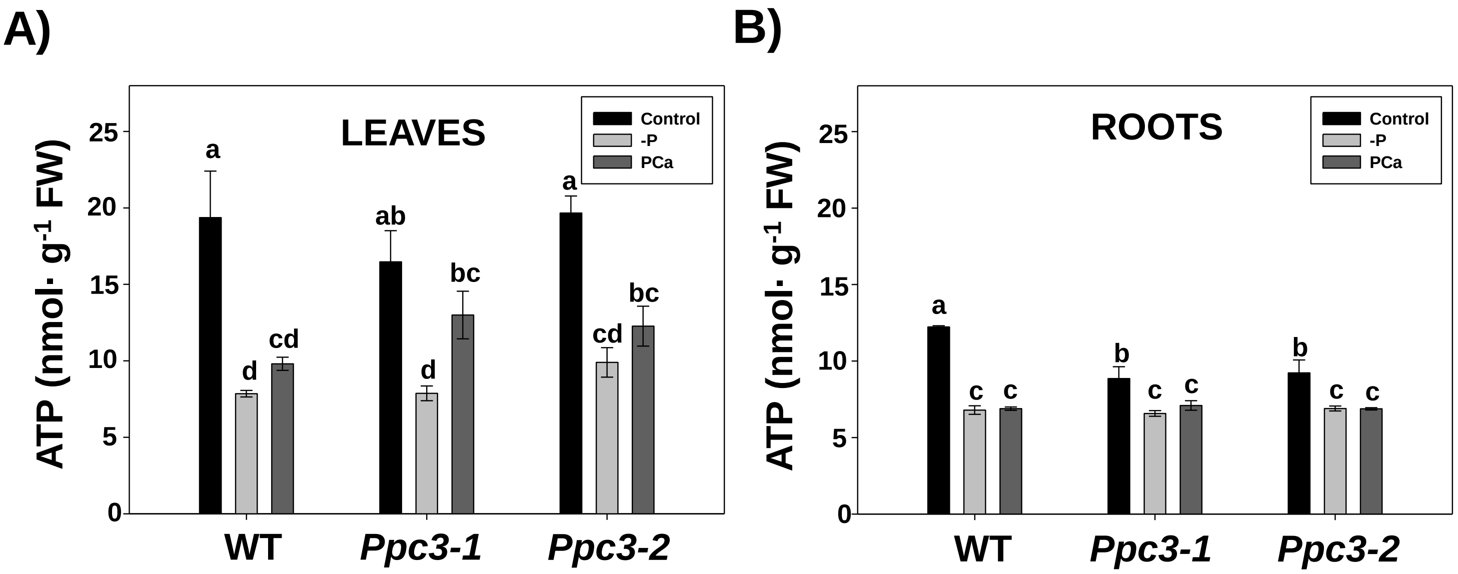

Supplemental Fig. S8. Effect of phosphate starvation on ATP levels in leaves and roots from WT and *Ppc3* plants. ATP levels measured in **(A)** leaves and **(B)** roots from sorghum plants. Bars indicate mean ± SE (n =3). Columns that do not have a common letter are significantly different by Duncan's multiple range test (P < 0.05).
