# Supplementary material for: Unravelling the Significance of Phosphoenolpyruvate Carboxylase in Phosphate Starvation Responses
Source: Plant Cell Environ. 2025 Sep 24;49(1):177–92. doi: 10.1111/pce.70204 (PMC12675990; doi:10.1111/pce.70204)
Supplement: Supplementary file 2 — Supplemental table S1: List of primers used in this work. Nucleotides in red spam an exon‐exon junction. [file PCE-49-177-s001.docx]

**Supplemental table S1. List of primers used in this work.** Nucleotides in red spam an exon-exon junction.

| Gene | Access numbre | Orientation | Sequence |
| --- | --- | --- | --- |
| Sb*actin* |  | F | 5’-TCACCATCGGGGCAGAG-3’ |
|  |  | R | 5’-GGGAGGCAAGGATGGAC-3’ |
| Sb*PPC1* | Sb10g021330 | F | 5’-CAGCTTCGTTCGCGCTTCCC-3’ |
|  |  | R | 5’-TCGTAGCACTCCTGGACAAATTCG-3’ |
| Sb*PPC2* | Sb02g021090 | F | 5’- CCGCCTCGCAACACCTGAAACA -3’ |
|  |  | R | 5’- ACCGGGAGGTGGAACCGTGT-3’ |
| Sb*PPC3* | Sb04g008720 | F | 5’- TGTTGAACAGTTTCTGGAACCTCTT -3’ |
|  |  | R | 5’-GCTTCACAAGGGCAAGCCCAAAAG-3’ |
| Sb*PPC4* | Sb07g014960 | F | 5’- TGAGCTTCGGGCACAAGCAGATG-3’ |
|  |  | R | 5’- GCTCCAAAGGCTCTAAGAACTGCTC -3’ |
| SbPPC5 | Sb03g035090 | F | 5’- GGACATCAGGGAGACGGTGCAAGA -3’ |
|  |  | R | 5’-ACCGGGAGGTGGAACCGTGT-3’ |
| SbPPC6 | Sb03g008410 | F | 5’- ACCACAGGGTTCGTAAAGCACGC -3’ |
|  |  | R | 5’-TGGCTTAGATCAGGGCGACCGTT-3’ |
| Sb*PHT1* | Sb01g046890 | F | 5’- GGCCAAGGTGCTCAAGAAG -3’ |
|  |  | R | 5’- GGAGGAACTGCACCGAGAAG -3’ |
| Sb*PX1* | Sb10g185200 | F | 5’- TGTGAAGCAGTGTGAAGCCA -3’ |
|  |  | R | 5’- CAGTGACCTAAGGGCTGCAA -3’ |
